# Comparative study of single-nucleotide polymorphism array and next generation sequencing based strategies on triploid identification in preimplantation genetic diagnosis and screen

**DOI:** 10.18632/oncotarget.13247

**Published:** 2016-11-09

**Authors:** Jiawei Xu, Wenbin Niu, Zhaofeng Peng, Xiao Bao, Meixiang Zhang, Linlin Wang, Linqing Du, Nan Zhang, Yingpu Sun

**Affiliations:** ^1^ The First Affiliated Hospital of Zhengzhou University, Centre for Reproductive Medicine, Zhengzhou, Henan 450000, China

**Keywords:** triploidy, SNP array, next generation sequencing, MALBAC, preimplantation genetic diagnosis and screen

## Abstract

Triploidy occurred about 2-3% in human pregnancies and contributed to approximately 15% of chromosomally caused human early miscarriage. It is essential for preimplantation genetic diagnosis and screen to distinct triploidy sensitively. Here, we performed comparative investigations between MALBAC-NGS and MDA-SNP array sensitivity on triploidy detection. Self-correction and reference-correction algorism were used to analyze the NGS data. We identified 5 triploid embryos in 1198 embryos of 218 PGD and PGS cycles using MDA-SNP array, the rate of tripoidy was 4.17‰ in PGS and PGD patients. Our results indicated that the MDA-SNP array was sensitive to digyny and diandry triploidy, MALBAC-NGS combined with self and reference genome correction strategies analyze were not sensitive to detect triploidy. Our study demonstrated that triploidy occurred at 4.17‰ in PGD and PGS, MDA-SNP array could successfully identify triploidy in PGD and PGS and genomic DNA. MALBAC-NGS combined with self and reference genome correction strategies were not sensitive to triploidy.

## INTRODUCTION

Triploidy is an abnormal chromosome kayotype, which occurred about 2-3% in human pregnancies [1] and contributed to approximately 15% of chromosomally caused human early miscarriage. The biological mechanism of triploidy may be of either digyny (one more haploid got from mother) or diandry (one more haploid got from father), and digynic triploidy predominates in fetuses leading to about 50–60% of early triploidy spontaneous pregnancy loss [2–5]. Although genomic imprinting or whole genome gene expression disturbed resulted to triploidy often early spontaneous abortion, triploidy still occasionally could develop to fetal or newborn period with the birth of an abnormal fetus or infants [6]. And assessment of embryonic phenotype with parental origin showed no correlation between the phenotype of the embryo and parental origin of the extra haploid set in triploid pregnancy [7].

Three pronucleis (3PN) embryo formation is common during in vitro fertilization (IVF), and is believed that polyspermic fertilization or oocyte-derived meiotic failure lead to triploid [8, 9]. Those patients with advanced maternal age or severe sperm abnormalities significant increased the incidence of triploidy fertilization during IVF [10]. Intracytoplasmic sperm injection (ICSI) essentially excluded dispermic triploidy but cannot prevent oocyte-induced triploid, such as the second polar boby exhausted failure [11]. It had been reported that triploid embryos negatively associated with IVF pregnancy outcome [12]. ICSI was recommended in patients who would undergo treatment of premimplantation genetic diagnosis and screen (PGD/S) [13]. Previous study suggests that 3PN embryos formation in ICSI treatment was mainly due to nonextrusion of the second polar body [14], severe sperm abnormalities, oocyte aging and women who are high responders to gonadotropins may also contribute to this process [9, 10, 15]. Sometimes 3PN embryos could not be observed due to fusion of pronuclear during IVF, hence, it was essential to consideration the formation and detectable of triploid embryos during PGD/S.

PGD/S was used for chromosome structure abnormality carrier patients, single gene mutation carrier patients, advanced maternal age couples, recurrent implantation failure and recurrent miscarriage patients to screen embryos genetic condition prior to transfer [16], along with single cell amplification technology developed, genome-wide technologies such as array-CGH, single-nucleotide polymorphism (SNP) array and next generation sequencing (NGS) are applied to PGD/S [17–19]. All of these three methods can successfully detect chromosome imbalances in embryos, also providing extra benefit of simultaneous aneuploidy screen of all 24 chromosomes [20, 21]. NGS was becoming more and more popular in PGD/S for lower cost, higher resolution and providing opportunity to simultaneously analyze single-gene disorders and genome-wide chromosome imbalance diagnosis and screen [22–24]. MDA and MALBAC, two main powerful single cell amplification methods were widely used in PGD/S, have performed high coverage and accuracy of whole genome [25, 26]. SNP based noninvasive prenatal testing (NIPT) have distinguished triploid pregnancy, and NGS based NIPT together with NATUS algorithm analyzing sequencing data identified triploid pregnancy using cfDNA in maternal blood [27, 28]. Although PGD/S has increased the pregnancy rate, no studies explore the ability on triploid embryos detection between SNP array and NGS technologies in PGD/S.

Here in our study, we systematically compared SNP array and NGS on detection chromosome deletion, duplication, uniparental disomy, mosaic and triploidy. We firstly identified triploid embryos using SNP array and compared it with NGS during PGD/S, our results indicated that present NGS based PGD/S procedures were unable to detect triploid embryos but SNP array can successfully distinguished triploidy.

## RESULTS

### Triploid embryos during PGD/S using MDA-SNP array

**Figure 1:**
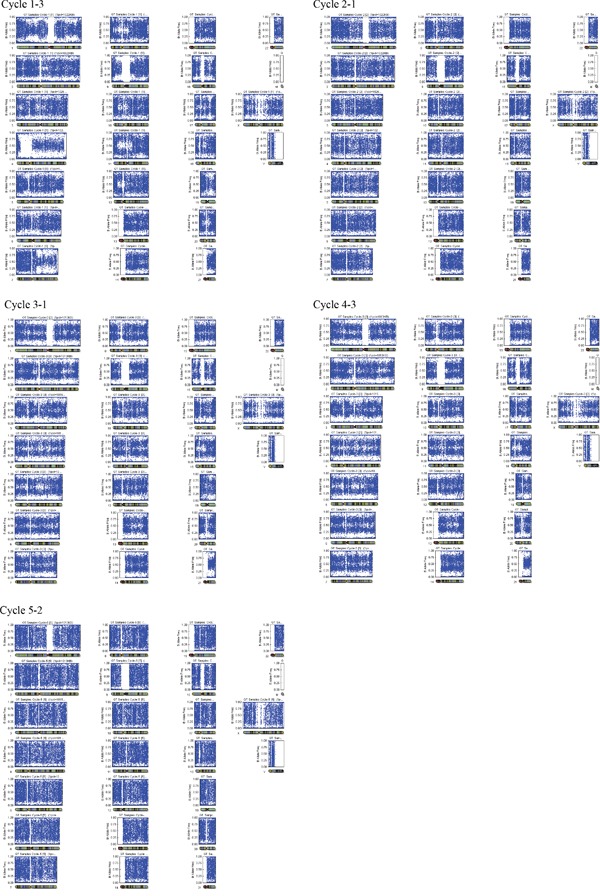
Triploidy detection during PGD/S using SNP array Case 1-3: arr (1-22)×3,(X)×3; Case 2-1: arr (1-22)×3,(X)×3; Case 3-1: arr (1-22)×3,(X)×3; Case 4-3: arr (1-22)×3,(X)×3; Case5-2: arr (1-22)×3,(X)×3.

We have detected 5 triploid embryos in 1198 embryos of 218 PGD/S cycles using MDA-SNP array (Table 1 and Figure 1). The rate was 4.17‰, which is lower than previous reported human triploid pregnancy rate 2-3% [1]. These triploid embryos in 5 PGD cycles with three chromosome translocation and two robertsonian translocation. All the triploidy detected were arr (1-22) ×3, (X) ×3 (Table 1). We didn't detect any arr (1-22) ×3, XYY and arr (1-22) ×3, XXY karyotype.

**Table 1:** Clinical characteristic of Triploid detected PGD/S cycles using SNP array

ID	F_Age	F_K	M_Age	M_K	N(Oocyte)	N(MII)	Days	E_ID	E_ Score	Embryo SNP Karyotype
Cycle 1	28	46,XX	24	46,XY,t(4,7)(p1 6,q22)	11	7	5	1	3BB	arr 4p(p15.31→qter)×3,7p(pter→q22.1)×1,(X)×2
							5	2	3BB	Amplification Failure
							**5**	**3**	**3BB**	**arr (1-22)×3,(X)×3**
Cycle 2	27	46,XX	27	45,XY,rob(13;1 4)(q10;q10)	9	6	**5**	**1**	**3BC**	**arr (1-22)×3,(X)×3**
							5	2	2BB	arr 21(pter→qter)×1,(X)×2
							5	3	3BB	arr (1-22)×2,(X)×2
							5	4	4BC	arr (1-22)×2,(XY)×1
Cycle 3	34	46,XX	38	45,XY,rob(14;2 1)(q10;q10)	16	16	**5**	**1**	**3BB**	**arr (1-22)×3,(X)×3**
							5	2	4AB	arr (1-22)×2,(XY)×1
							5	3	6BB	arr (1-22)×2,(XY)×1
Cycle 4	29	46,XX,t(9,1 7)(p13,q12)	31	46,XY	21	19	5	1	3BB	arr (1-22)×2,(XY)×1
							5	2	4BB	arr 9q(q13→qter)×3,17p-,(X)×2
							**5**	**3**	**2BB**	**arr (1-22)×3,(X)×3**
							5	4	2BB	arr 9(pter→qter)×3,22 (pter→qter)×1, 17p-,(XY)×1
							5	5	2BC	arr 19p(p13.2→pter)× 1,(X)×2
Cycle 5	27	46,XX,t(11;1 5)(p15.4;q25)	27	46,XY	15	14	5	1	3BB	arr (1-22)×2,(X)×2
							**5**	**2**	**2BB**	**arr (1-22)×3,(X)×3**
							5	3	3BB	arr 4q(q33→qter)×1, (X)×2
							6	4	5BB	arr 16(pter→qter)×1, (X)×2
							6	5	5BB	arr (1-22)×2, (X)×2
							6	6	6BB	arr (1-22)×2,(X)×2

5 cycles identified triploidy were shown from 218 cycles.

**F_Age**: Female age, **F_K**: Female karyotype, **M_Ag**e: Male age, **M_K**: Male karyotype, **N(Oocyte)**: Number of retrieve oocyte, **N(MII)**: Number of MII oocyte, **E_ID**: Embryo_ID, **E_ Score**: Embryo Score.

### Comparative MDA-SNP array and MALBAC-NGS during copy number variance screen

We firstly compared the SNP array and NGS on detection copy number variance. We diluted DNA from previously known SNP karyotype missed abortion chorionic tissues then whole genome amplification using MALBAC. The amplification product were sequenced and analyzed, 46 samples previously known SNP array karyotype including duplication, deletion, mosaics and uniparental disomy (UPD), the results of SNP array and NGS were highly in accordance (Table 2). The NGS is sensitive to duplication, deletion and mosaics, but UPD couldn't be identified. Furthermore, NGS could identify more copy number variance than SNP array and is powerful on detection low percentage mosaics.

**Table 2:** Comprehensive comparison of MDA based SNP array and MALBAC-NGS

ID.	SNP array karyotype	NGS Karyotype
1	arr 21(pter→qter)×3,(X)×2	47,XX,+21(×3)
2	arr 10(pter→qter)×3[mos 20],(X)×2	46,XX,+10q(q21.3→qter,~64M,×3,mos,~40%)
3	arr 14(pter→qter)×3,(X)×2	47,XX,+4q(q28.1→q32.2,~38M,×3,mos),+14(×3),-17(p11.2→q21.33,~30M,×1,mos),-19q(q13.11→qter,~23M,×1,mos)
4	arr (1-22) ×2,(X)×2	46,XX
5	arr (1-22) ×2,(X)×2	46,XX
6	**arr21(pter→qter)×1,UPD(6p21.32→6p22.1)(27226965-35943151)×2 hmz,(XY)×1**	**45,XY,-21(×1)**
7	arr (1-22)×2,(X)×2	46,XX
8	arr 16(pter→qter)×3,(X)×2	47,XX,+16(×3)
9	arr 20(pter→qter)×3, (X)×2	47,XX,+20(×3)
10	arr 8p(pter→p12)×1,8q+[40]/8p(pter→p12)×1,(XY)×1	46,XY,-8p(pter→p12,~32M,×1)
11	arr 14(pter→qter)×3, (XY)×1	47,XY,+14(×3)
12	arr 4(pter→qter)×3, (X)×2	47,XX,+4(×3)
13	arr 7(pter→qter)×3, (XY)×1	47,XY,+7(×3)
14	arr 16(pter→qter)×3,(XY)×1	47,XY,+16(×3)
15	arr 16(pter→qter)×3,(XY)×1	47,XY,+16(×3)
16	arr (1-22)×2,(XY)×1	46,XY
17	46,XX,dup(1)(q32.1→qter)[100],12p+[15], dup(18)(q21.32→qter)[20]	46,XX,-1q(q32.2→qter,~38M,×1,mos),+18q(q21.31→qter,~21M,×3,mos)
18	arr 1q(q44)×1,8q(q24.22→qter)×3,(XY)×1	46,XY,+8q(q24.21→qter,~15M,×3)
19	arr 8(pter→qter)×3,20(pter→qter)×3,(X)×2	48,XX,+18(×3),+20(×3)
20	arr (1-22)×2,(XY)×1	46,XY
21	arr 22(pter→qter)×3, 20(pter→qter)×3[18]/20(pter→qter)×3[82] (XY)×1	47,XY,+22(×3)
22	arr 8p(p22→pter)×1,(X)×2	46,XX,-8p(pter→p23.1,~12M,×1)
23	arr 16(pter→qter)×3,(XY)×1	47,XY,+16(×3)
24	arr 15(pter→qter),(X	47,XX,+15(×3)
25	arr 15(q24.1-qter)×3,(XY)×1	46,XY,+15q(q24.1→qter,~28M,×3)
26	arr 22(pter→qter)×3,(XY)×1	47,XY,+22(×3)
27	arr 3(pter→qter)×3,(XY)×1	47,XY,+3(×3)
28	arr (1-22)×2,(X)×2	46,XX,+8q(q24.13→qter,~20M,×3,mos,~40%)
29	arr 13(pter→qter)×3,22(pter→qter)×3,(XY)×1	47,XY,+13(×3)
30	arr 18(pter→qter)×3,(XY)×1	47,XY,+18(×3)
31	arr 16(pter→qter)×3,(XY)×1	47,XY,+16(×3)
32	arr 19(p12)(22130311-23202379)×3,(X)×2	46,XX,+13q(q31.1→qter,~30M,×3,mos,~30%)
33	arr 11q(q22.3→q25)×3,11q(q25)×1,(XY)×1	46,XY,+11q(q22.3→q24.2,~19M,×3)
34	arr 22(pter→qter)×3,(X)×2	47,XX,+22(×3)
35	arr 22(pter→qter)×3,(X)×2	47,XX,+22(×3)
36	arr 16 arr 16(pter→qter)×3,(X)×2	47,XX,+16(×3)
37	arr 5q(q23.1→qter)×3,10(q26.2→q26.3)×1,(X)×2	46,XX,+5q(q22.3→qter,~65M,×3)
38	arr 6(pter→qter)×3,(X)×2	47,XX,+6(×3)
39	arr 13(pter→qter)×3,(XY)×1	47,XY,+13(×3)
40	arr 16(pter→qter)×3,(XY)×1	47,XY,+16(×3)
41	arr 18(pter→qter)×3,(X)×2	47,XX,+18(×3)
42	46,XX,dup(8)(q12.1→q21.1)30%, dup(19)(q13.12→qter)30%,dup(18)(q21.2→q22.1),del(18)(q22.1→qter)	46,XX,+18q(q11.2→q21.2,~28M,×3,mos),-18q(q21.32→qter,~20M,×1)
43	arr 22(pter→qter)×3,(X)×2	47,XX,+22(×3)
44	arr 4q(q28.3→qter)×3[mos 15],(X)×2	46,XX,4q(q28.3→qter)×3[mos 30]
45	arr 6(pter→qter)×3,(X)×2	47,XX,+6(×3),-13q(q14.3→q22.1,~21M,×1,mos)
46	arr 16(pter→qter)×3,(X)×2	47,XX,+16(×3)

### Digyny and diandry triploidy detection using MDA-SNP array

**Figure 2:**
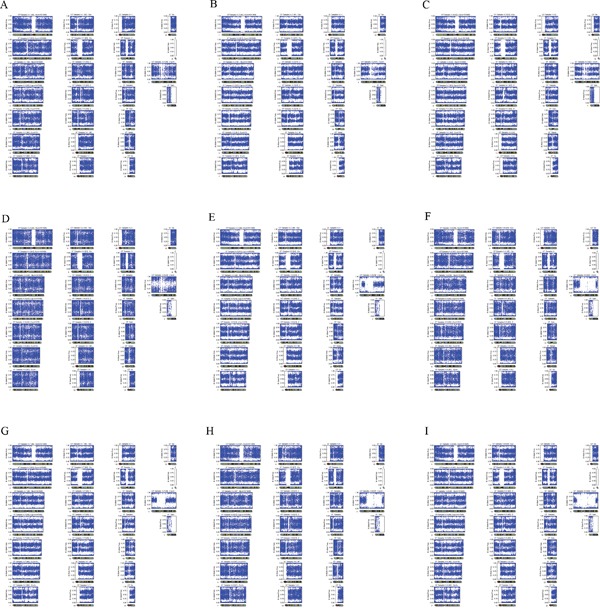
Three types of triploid identification of chorionic tissues using MDA based SNP array on single cell level **A-C.** arr (1-22)×3,(X)×3; **D.** 69,XYY, **E-I.** 69,XXY.

Our previous data detected triploidy in five PGD cycles, however, these only identified arr (1-22)×3,(X)×3, however, whether the arr (1-22) ×3, (XXY)×1 and arr (1-22) ×3, (XYY) ×1 could be detected or not in single cell lever using MDA based SNP array is still unknown. Here we system investigated the sensitivity of MDA based SNP array for the digyny and diandry triploidy on single cell lever. Our results showed MDA based SNP array could completely identify the arr (1-22)×3,(X)×3, arr (1-22) ×3, (XXY)×1 and arr (1-22) ×3, (XYY) ×1 (Figure 2), indicating that all types of triploidy would be detected during PGD/S.

### Digyny and diandry triploidy detection using MALBAC-NGS

**Figure 3:**
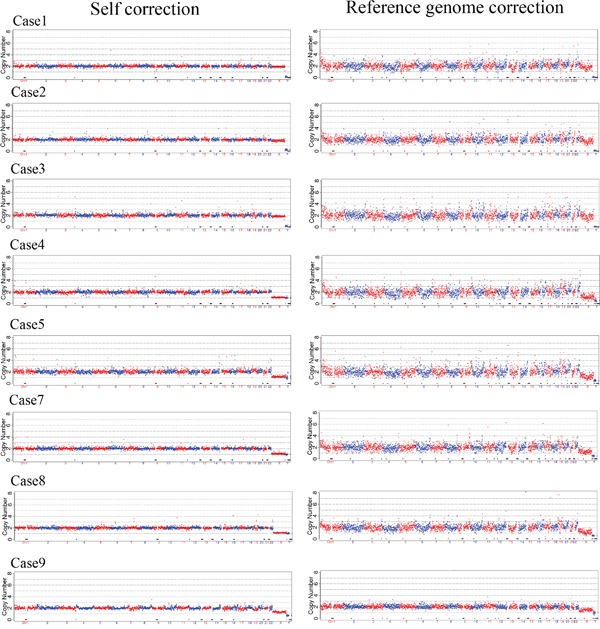
Self and reference genome correction of triploid chorionic tissue using NGS

We selected 9 previous known triploids SNP array karotype to validate using NGS. The 9 triploid SNP array kayotype were shown in Supplementary Figure 1, one was amplification failure in our study. The basic information of 9 sample data was shown in Supplementary Table 1. After sequencing, we firstly using reference karyotype methods to analyzed the data, and we found that NGS could not detect triploid for it considered the triploid as normal diploid (Supplementary Table 2). Then we using self correction method to analyze the data, this could not detect the triploid karyotype (See Figure 3). The unique reads of NGS data were ranging from 1.23M to 4.84Mb, both the low and high coverage didn't identify the triploidy in our study. Our results indicated that both self and reference correction may not be able to detect the triploid, mostly because both these methods calculated the reads of 24 chromosome falling into the continuous sliding widows on human reference genome.

## DISCUSSION

Triploidy is an abnormal chromosome kayotype, which occurred at very low percent in human pregnancies, this is the first time comprehensive analyzed the triploidy detection using SNP array and NGS in PGD/S and missed abortion chorionic tissues. Our results indicated that the rate of tripoidy was 4.17′ in PGD/S patients. In our study, we comprehensive compared the MDA based SNP array and MALBAC based NGS for triploid detection on single cell lever. And we concluded that SNP array could detect triploidy, while the present strategies of NGS are not sensitive to triploidy detection.

PGD and PGS were used to detect the aneuploidy and pathological gene carrier embryos and has been used widely in clinical [19], which had been proved to increase the pregnancy rate of chromosome structure abnormality carrier patients, advanced maternal age, mendelian diseases and recurrent miscarriage patients [17, 29, 30]. Previous data showed that PGD and PGS were good strategy for single embryo transfer which would reduce the multiple pregnancy rate without affecting the clinical outcomes and take baby home rate [31]. The accuracy and coverage of PGD and PGS technology are critical factors for its clinical applications, such as all aneuploidy kayotypes were successfully detected. SNP-array PGD was reported that may increase the clinical pregnancy outcome of translocation carriers [18]. However, only matched cohort studies relating to patients of advanced maternal age, recurrent miscarriage and implantation failure, limiting the ability to draw meaningful conclusions [32]. Triploidy as an abnormal embryo karyotype can occur at low rate in human spontaneous conception and IVF, it is negative correlation with pregnancy rate in IVF [12]. In our study, our data showed that both SNP array and NGS were sensitive to chromosome duplication, deletion and mosaic, while NGS could not identify uniparental disomy and not sensitive to triploidy.

In our study, both self and reference genome correction were used to analyze the NGS data of triploid DNA of chorionic tissues, and we found these two methods could not identify triploidy. The present bioinformatics algorithms mainly calculate the reads falling into the “continues windows” usually ranging from 1K to 1M on each chromosome, it is able to identify the reads number of the chromosome deletion, duplication and mosaic as shown in our study, but it ignores the SNPs and could not detect triploidy. Triploidy usually leads to the SNPs three nucleotides heterozygosis and the homozygous/heterozygosis was 1:2, SNP array well distinguishes the SNPs and well detects the triploidy even after the single cell whole genome amplification, while NGS needs to sequence deeper and much powerful bioinformatics algorisms to analyze the data. Although the NGS is much more powerful on detection micro duplication and deletion, it is weaker than SNP on detection triplody. SNP-based approach detected the relative distributions of alleles at polymorphic loci and does not require a reference chromosome for comparison [28]. Although MALBAC has increased the whole genome coverage rate, the triploidy still enlarge allele dropout rate for every chromosome of triploidy has same frequency to be amplification. Together with the genome recombination of triploidy will increase the difficulty of identification of triploidy. NATUS algorithm was used to distinguish the triploidy fetus using cfDNA from maternal blood, however it could not identify the SNPs precisely, for the allele dropout of single cell amplification [28]. Further study and strategies are essential to be developed to identify the triploidy using NGS. Time-lapse cooperated with NGS would be a better solution to decrease the insensitivity to triploid.

## MATERIALS AND METHODS

### PGD and PGS patients controlled ovarian stimulation and embryos biopsy

The controlled ovarian stimulation (COS) of all the patients underwent PGD and PGS were carried out in a long protocol, GnRH analogues was used for pituitary desensitisation, together with human menopausal gonadotrophins (hMG) or recombinant FSH. The starting dose of gonadotrophins for PGD and PGS patients was determined according to the patient's age, BMI and/or previous response to ovarian stimulation (range from 75 to 300 IU QD). Human chorionic gonadotrophin (hCG) was administered when at least 60% follicles above 16 mm mean diameter and the biggest under 22 mm mean diameter were seen when transvaginal ultrasound scan. Transvaginal ultrasound-guided and vacuum take-off oocytes collection was scheduled 36 h after hCG administration. Regardless of the sperm quality, Intra Cytoplasmic Sperm Injection (ICSI) was performed rather than IVF to prevent DNA contamination with sperm and cumulus cell's DNA during PGD and PGS. Fertilization was assessed 17–20 h after ICSI and embryo cleavage was recorded every 24 h. Embryo biopsy was performed on day 5 or 6 at blastocyst stage. All patients were informed consents in our study [33].

### Single cell multiple displacement amplification (MDA) and SNP array

Multiple Displacement Amplification (MDA) was used for single cell amplification in SNP array. Briefly, REPLI-g Single Cell Kit (QIAGEN, 150345) was used for single cell amplification, single cell was seeding in 4.5μl phosphate-buffered saline, and then was lysised using 3μl DTT and DLB in incubator for 10 min at 65°C. After incubation, 3μl Stop Solution was added in the mix. The amplification mix was prepared and 40μl including 2μl REPLI-g sc DNA polymerase and 29μl REPLI-g sc Reaction Buffer was added to each tube. Then the mixture was incubated in incubator for 8h at 30°C following 65°C for 3 min to inactivate the REPLI-g sc DNA polymerase. The single cell amplification mix was stored at −20°C until for SNP array. The DNA of previous triploidy detected using SNP was diluted and amplification as described above. All the procedures were under the direction of Illumina human cyto12 microarray with minor modification in our center.

### Single cell multiple annealing and looping based amplification cycles (MALBAC) and next generation sequencing (NGS)

Multiple Annealing and Looping Based Amplification Cycles (MALBAC) was used for whole genome amplification then high throughput sequencing. The DNA from previously SNP array results were quantified again with Qubit and then were diluted to 10pg for single cell amplification using YK015CHR (Yikon Genomics). Firstly the DNA was diluted into 4.5μl lysis buffer and lysis enzyme, the amplification products were fragmentation and ligated adapter, then PCR and magnetic purification according to the user manual. The barcoded DNA was sequenced using Hiseq 2500 Rapid 1×50 mode in our center.

### Single cell and multi cell SNP array data analysis

The SNP array data was analyzed using GenomeStudio Software (2011, Illumina). B allele frequency and Log R ratio was used to analyzed the genotype. The copy number variance was called using KaryoStudio Software v1.4. The uniparental disomy was reported by both KaryoStudio and GenomeStudio.

### NGS data analysis using self correction and reference karyotype

The raw data (in.bcl format) was demultiplexed and converted to FASTQ format using a perl script configureBclToFastq.pl in CASAVA 1.8.4 package based on the sample-sheet information. Illumina adaptors, low quality bases (bases with quality score less than 20) and MALBAC primers were removed from the FASTQ file using Trimmomatic [34].

High quality reads were mapped to hg19 reference genome using BWA with default parameters [35]. The mapped reads were sorted and converted to binary format using SamtoBam.jar in Picard package. Unique mapped reads were extract from the alignment reads (.bam file). Then the whole reference genome was divided into non-overlapping observation windows (bins) with size of 1Mb. Reads number, GC content were calculated in each bin. GC bias correction was processed for every 1% GC content. The GC corrected relative reads number (RRN) of each bin was corrected by the reference training set [36]. 500 normal chromosome samples were prepared as reference training set, as well as self-correction were used to analyze the data. We use R programming language to graph the final RRN of each bin to visualize copy number variations.

## SUPPLEMENTARY MATERIALS FIGURE AND TABLES


