# Supplementary material for: Comparative study of single-nucleotide polymorphism array and next generation sequencing based strategies on triploid identification in preimplantation genetic diagnosis and screen
Source: Oncotarget. 2016 Nov 9;7(49):81839–48. doi: 10.18632/oncotarget.13247 (PMC5348434; doi:10.18632/oncotarget.13247)
Supplement: Supplementary file 1 [file oncotarget-07-81839-s001.pdf]

Comparative study of single-nucleotide polymorphism array and next generation sequencing based strategies on triploid identification in preimplantation genetic diagnosis and screen

SUPPLEMENTARY FIGURE AND TABLES

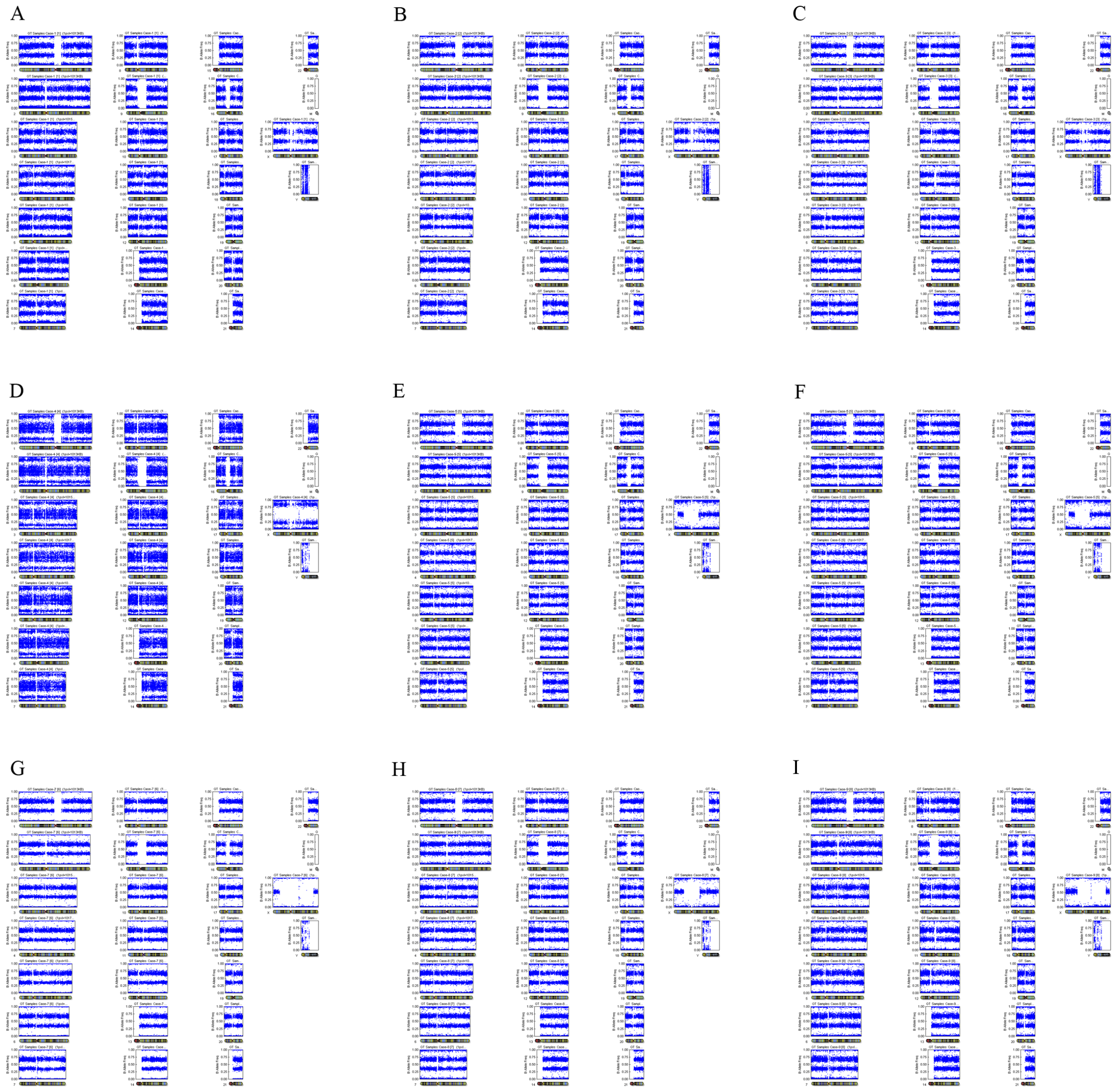

Supplementary Figure S1: Digyny and diandry triploidy detection using SNP array. A-C. arr (1-22)×3,(X)×3; **D.** 69,XYY, **E-I.** 69,XXY.

Supplementary Table S1: Basic statistical of MALBAC-NGS Data

ID	Raw_reads	GC%	HQRs(HQRs/Raw reads)	MRs(MRs/HGRs)	UMRs(UMRs/Raw reads)
S1	2,095,199	47	1,847,495(88.18%)	1700465(92.04%)	1,423,957(67.96%)
S2	1,659,345	48	1,476,884(89%)	1344292(91.02%)	1,114,163(67.14%)
S3	1,817,737	48	1,612,165(88.69%)	1477250(91.63%)	1,258,078(69.21%)
S4	1,046,231	48	934,879(89.36%)	834412(89.25%)	656,599(62.46%)
S5	1,533,121	48	1,377,612(89.86%)	1259588(91.43%)	1,013,801(66.13%)
S6	65,951	48	58,857(89.24%)	52465(89.14%)	41,396(62.77%)
S7	1,703,921	48	1,541,965(90.5%)	1418053(91.96%)	1,108,233(65.04%)
S8	1,492,861	48	1339568(89.73%)	1227843(91.66%)	947,085(63.44%)
S9	6,501,280	42	5,490,095(84.45%)	4836706(88.1%)	3,968,259(61.04%)

HQRs: high quality reads; MRs: Mapped_reads; UMRs: Unique_mapped_reads.

Supplementary Table S2: Comparison of SNP array and NGS on detection of Triploid

ID	SNP karyotype	Putative Triploidy type	NGS karyotype (Self correction)	NGS karyotype (Relative correction)
1	arr (1-22)×3,(X)×3	Digynic/Diandry	46,XX	46,XX
2	arr (1-22)×3,(X)×3	Digynic/Diandry	46,XX	46,XX
3	arr (1-22)×3,(X)×3	Digynic/Diandry	46,XX	46,XX
4	arr (1-22)×3,(XXY)×1	Digynic/Diandry	46,XY	46,XY
5	arr (1-22)×3,(XXY)×1	Digynic/Diandry	46,XY	46,XY
6	arr (1-22)×3,(XXY)×1	Digynic/Diandry	Amplification failure	Amplification failure
7	arr (1-22)×3,(XYY)×1	Diandry	46,XY	46,XY
8	arr (1-22)×3,(XYY)×1	Diandry	46,XY	46,XY
9	arr (1-22)×3,(XXY)×1	Diandry	46,XY	46,XY
